# Dataset of aerial photographs acquired with UAV using a multispectral (green, red and near-infrared) camera for cherry tomato (*Solanum lycopersicum* var. *cerasiforme*) monitoring

**DOI:** 10.1016/j.dib.2024.111256

**Published:** 2024-12-24

**Authors:** Osiris Chávez-Martínez, Sergio Alberto Monjardin-Armenta, Jesús Gabriel Rangel-Peraza, Zuriel Dathan Mora-Felix, Antonio Jesus Sanhouse-García

**Affiliations:** a Universidad Autónoma de Sinaloa, Facultad de Ciencias de la Tierra y el Espacio. Circuito Interior Oriente SN, Cd Universitaria, 80040 Culiacán, Sinaloa, Mexico; b Tecnológico Nacional de México/Instituto Tecnológico de Culiacán, División de Estudios de Posgrado e Investigación, Juan de Dios Batíz 310. Col. Guadalupe, 80220 Culiacán, Sinaloa, Mexico; c Laboratorio Nacional CONAHCYT de Tecnologías de la Información Geoespacial para los Sistemas Socioecológicos Resilientes (LaNCTIGeSSR), clave 89. Cerro de Coatepec, Ciudad Universitaria, 50110 Toluca de Lerdo, Mexico

**Keywords:** High-resolution multispectral images dataset, UAV DJI Phantom 4 Pro, Mapir survey 3W multispectral camera, Cherry tomato crop, Precision agriculture

## Abstract

A dataset of aerial photographs acquired with an Unmanned Aerial Vehicle (UAV) DJI Phantom 4 Pro is presented for monitoring a cherry tomato (*Solanum lycopersicum* var. *cerasiforme*) crop in Navolato, Mexico. Seven photogrammetric flights were carried out to assess the plant growth using a Mapir Survey 3W multispectral camera. Multispectral images with an approximate spatial resolution of 1.83 cm/px were obtained in each photogrammetric flight. These images were acquired every 15 days starting on October 15, 2021, and ending on January 23, 2022. The dataset contains the radiometrically calibrated images of the tomato crop divided into 2 open field parcels. The dataset also includes the processed photogrammetric products (ortho-mosaics) using a binary mask to exclude the soil from the plant area. The dataset was originally acquired to assess plant growth, stress levels, and overall crop health. However, this multispectral imagery dataset can also have various uses, such as creating training datasets with accurate labels or classes which can then be used to develop, train, and/or validate machine learning algorithms for image classification, object detection tasks, or change detection analysis.

Specifications Table

**Table:** 

Subject	Precision agriculture
Specific subject area	Vegetation Indices, change detection analysis.
Type of data	GRN (Green, Red, and Near Infrared) aerial imagery, images taken manually from the plants, orthomosaics, and plant classification (binary mask) in TIF format. Details about the photogrammetric flight conditions, including the date, time, temperature, and relative humidity, are also provided. In addition, some observations were made to identify the phenological stage of the plant (BBCH scale).
Data collection	The dataset images were collected using the DJI Phantom 4 Pro unmanned aerial vehicle at a flight speed of 14.1 m/s and a height of 40 m, equipped with a Mapir Survey 3W camera featuring a Sony ExmorR IMX117 12MP (Bayer RGB) sensor, GPS/GNSS ublox UBX-G7020-KT, 87° HFOV (19 mm) f/2.8 aperture lens with extremely low distortion (non-fisheye) glass lens, capturing at a resolution of 12 Mpx (4000 × 3000 px) in RAW (12-bit) and JPG (8-bit) formats, with a capture time of 1 s.
Data source location	Country: Mexico. State/municipality: Sinaloa, Navolato. Coordinates: 24° 40′ 35.05′′ y -107° 32′ 27.99′′
Data accessibility	Repository name: tomatodb Data identification number: 10.5061/dryad.63xsj3vbd Direct URL to data: https://datadryad.org/stash/share/Wq_X7QUyGryJ-ZnmgfwRn4MtOCr4VBm_MSnhF40sv_8#readme
Related research article	O. Chávez-Martínez, S.A. Monjardin-Armenta, J.G. Rangel-Peraza, A.J. Sanhouse-García, Z.D. Mora-Felix, W. Plata-Rocha, Use of different vegetation indices for the evaluation of the kinetics of the cherry tomato (*Solanum lycopersicum* var. *cerasiforme*) growth based on multispectral images by UAV, Open Agric 9 (2024). 10.1515/opag-2022-0357.

## 1 Value of the Data

• The tomato crop monitoring images were acquired as part of specific precision agriculture activities related to the assessment of plant health and crop stress by using vegetation indices [1].
• Multispectral imagery datasets were acquired at different time intervals to analyze the phenological stages of the tomato cherry crop. This analysis can help understand the growth patterns and maturity stages of plants, which is valuable for crop management and predicting yield.
• The imagery provided is a high-resolution dataset that can be used as ground truth for other studies. This imagery can also be used to validate or assess the quality, reliability, and accuracy of remote sensing products, such as land cover maps and vegetation indices, such as NDVI (Normalized Difference Vegetation Index), GNDVI (Green Normalized Difference Vegetation Index) and RVI (Ratio Vegetation Index). These indices contribute to monitoring changes in crops, identifying nutrient deficiencies [2,3], detecting pest infestations [4,5], and optimizing irrigation [6,7].
• The scientific community can use the high-resolution data to train and validate models for object detection, classification, or change detection tasks.
• An independent dataset with orthomosaics is presented, including an orthomosaic with a binary mask performed with a supervised classification. This mask was implemented to exclude the effect of soil. Therefore, the orthomosaic obtained only represents plant pixels. The provided orthomosaics can be implemented for more accurate monitoring of crops or to develop new classification methods.
• The imagery dataset has a total of seven measurement campaigns (7 photogrammetric flights), starting on October 15, 2021, and ending on January 23, 2022. Two parcels (T1 and T2) were considered for the monitoring of the cherry tomato crop (*Solanum lycopersicum* var. *cerasiforme*).

## 2 Background

Monitoring crop conditions and growth patterns includes direct observation and manual measurements [8] Crop conditions can be inspected visually by walking through the fields, looking for signs of plant health, such as coloration, leaf shape, size, and overall appearance [9]. Nutrient deficiencies, pest or disease infestations, or crop stress can be identified by visual inspection, but specific crop parameters can also be measured such as counting plants, measuring plant height, or assessing canopy density [10]. Soil samples at various depths and locations within the field are analyzed in the laboratory to determine nutrient deficiencies or imbalances, soil texture, organic matter content, and other relevant information [11].

The acquisition of data for monitoring a crop can be laborious and time-consuming when large-scale crop monitoring is carried out. Satellite imagery and aerial photography reduce the labor and time required for crop monitoring and provide a broader view of crop conditions over large areas. UAVs as imaging platforms have the advantage of providing greater control over temporal and spatial resolution across large areas [6,12]. Tomato crop datasets acquired by UAVs can provide accurate information with a resolution of a few centimeters [5,13]. UAV imagery complements this data by adding a broader perspective to the information obtained from the ground. High-resolution imagery is used to assess crop health and conditions, detect stress factors, identify disease or pest infestations, and make informed decisions about irrigation, and fertilization.

In this paper, an extensive tomato crop dataset is presented as part of specific precision agriculture activities related to the assessment of plant health and crop stress by using vegetation indices in northeastern Mexico [1]. Other studies have previously shown that UAV multispectral image datasets can be used for mapping and predicting various parameters of interest in agriculture [14], and effectively monitoring crop development and changes [15,16]. Similar datasets have been also used for training algorithms for image classification [17] and object detection and tracking [18], thereby optimizing agricultural resource management.

When the objects present in a dataset are spectrally different, the dataset can be used to train and validate models for object detection, classification, or change detection tasks. The supervised machine learning (ML) classifications, such as support vector machine (SVM), random forest (RF), and artificial neural network (ANN) have been used to classify the elements present in a vineyard [19]. Modica et al. [20] used four classification algorithms (K-Nearest Neighbors (KNN), Support Vector Machines (SVM), Random Forests (RF) and Normal Bayes (NB)) to discriminate crop, bare soil, shade, and weeds on Bergamot and Onion. Deng et al. [21] evaluated a methodology integrating an object-oriented method and a random forest (RF) algorithm for crop identification using multispectral UAV imagery. Senthilnath et al. [22] applied spectral-spatial classification algorithms (K-means, expectation maximization (EM) and self-organizing map (SOM)) for tomato detection under a Bayesian criterion to categorize and segment the image into two classes (tomatoes and non-tomatoes).

To address the growing need for accurate tomato parameter estimation from satellite imagery [22,23], this work provides a publicly available multispectral UAV imagery dataset of cherry tomatoes (*Solanum lycopersicum* var. *cerasiforme*). Acquired at multiple time intervals, this dataset can validate satellite remote sensing data and support advancements in agricultural and precision agriculture research.

## 3 Data Description

**Fig. 1:**
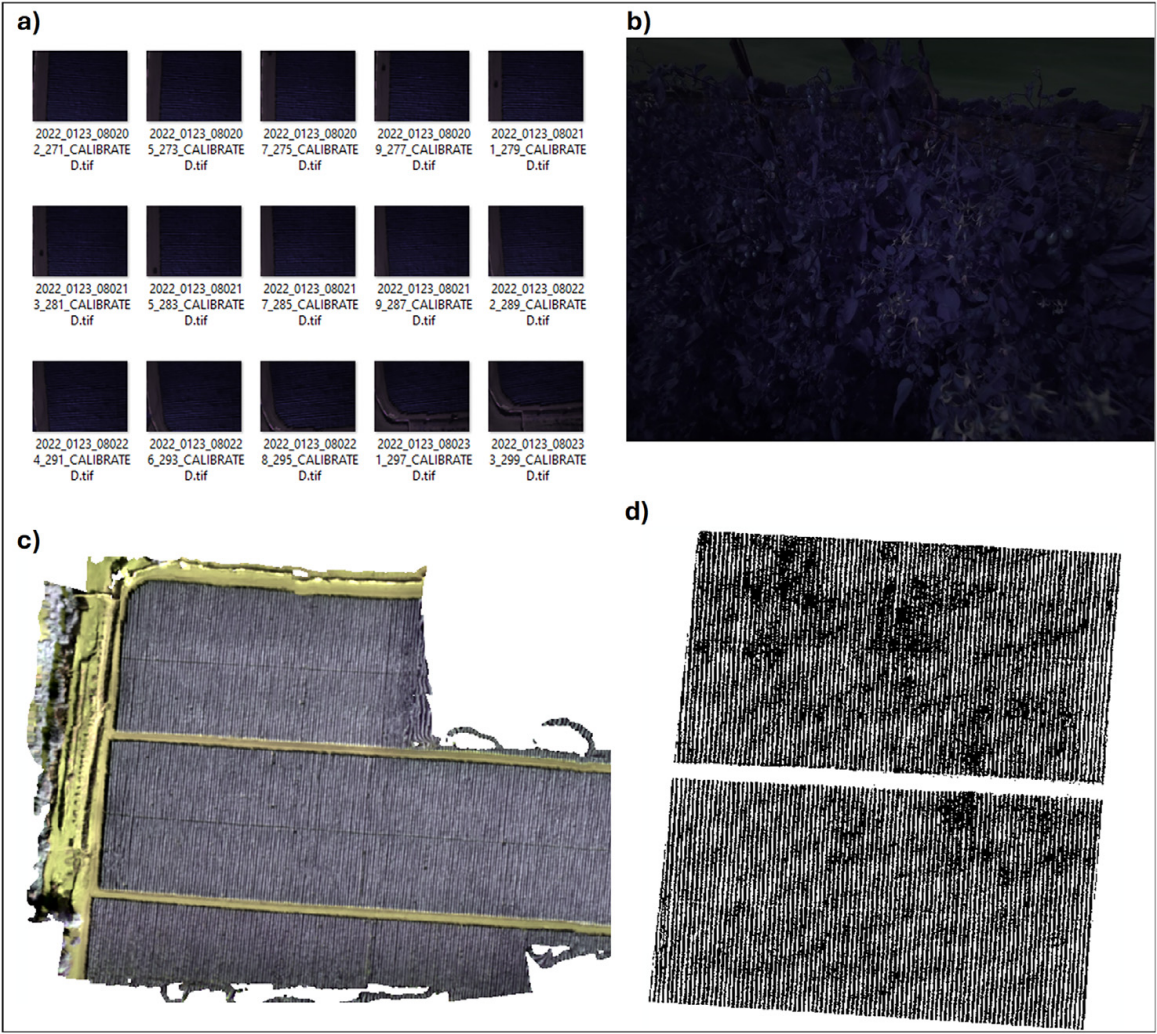
Examples of a) multispectral aerial images, b) images taken manually from the plants, c) ortomosaics and d) binary mask.

**Fig. 2:**
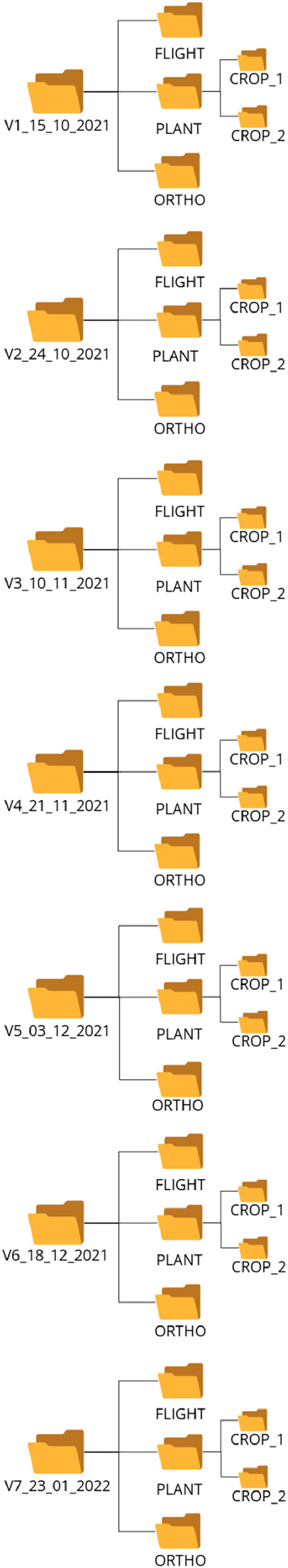
Dataset file organization. The dataset contains folders named by their sequential number and date (e.g., V1_15_10_2021), each representing a specific data acquisition session. Within each flight folder, three main subfolders are organized: FLIGHT, PLANT, and ORTHO. The FLIGHT and PLANT folders contain two subfolders, CROP_1 and CROP_2, corresponding to different crop (T1 and T2).

Fig. 2 shows the dataset organization. The folders are organized by flight date. Therefore, a total of seven folders can be found. Each folder contains three subfolders (FLIGHT, ORTHO, and PLANT). The FLIGHT folder contains the multispectral aerial images obtained from the photogrammetric flights (Fig. 1a). The ORTHO folder contains the orthomosaics obtained from image processing (Fig. 1c) and then when the binary mask was applied (Fig. 1d). The PLANT folder shows the multispectral images of the leaves and fruits of the crop (Fig. 1b). These images were taken manually at about 50 cm from the plant. Table 1 shows the particularities, format, and size of each file.

**Table 1:** Description and size of files.

Flight date	Remarks	Format	Size file
15/10/2021	745 calibrated images without removing the initial departure images	TIF	35.6 GB
15/10/2021	62 calibrated images were taken manually at about 50 cm from the plant		2.82 GB
24/10/2021	764 calibrated images without removing the initial departure images		33.9 GB
24/10/2021	49 calibrated images were taken manually at about 50 cm from the plant		2.03 GB
10/11/2021	492 calibrated images without removing the initial departure images		20.5 GB
10/11/2021	87 calibrated images were taken manually at about 50 cm from the plant		3.62 GB
21/11/2021	430 calibrated images without removing the initial departure images		18.4 GB
21/11/2021	97 calibrated images were taken manually at about 50 cm from the plant		4.08 GB
03/12/2021	404 calibrated images without removing the initial departure images		18.1 GB
03/12/2021	72 calibrated images were taken manually at about 50 cm from the plant.		3.20 GB
18/12/2021	400 calibrated images without removing the initial departure images		17.9 GB
18/12/2021	48 calibrated images were taken manually at about 50 cm from the plant.		2.07 GB
23/01/2022	420 calibrated images without removing the initial departure images		17.5 GB
23/01/2022	81 calibrated images taken manually at about 50 cm from the plant.		3.35 GB
-	7 orthomosaics without using binary mask		22.47 GB
-	7 orthomosaics excluding the ground. Image pixels only represent plants.		2.65
15/10/2021	Binary masks of the two treatments (T1 and T2).	TIF	5.45 MB
24/10/2021			5.13 MB
10/11/2021			5.13 MB
21/11/2021			4.46 MB
03/12/2021			6.04 MB
18/12/2021			4.91 MB
23/01/2022			4.76 MB
209 GB			

Table 1 shows the particularities, format, and size of each file. This dataset is in the DRYAD repository, an open data publishing platform that allows routine reuse of all research data. It can be accessed through the DOI or the URL indicated in the specification table. Once the URL is clicked, the website shows the dataset's title, the dataʼs download links, the README file, the abstract, and the description of the data structure.

## 4 Experimental Design, Materials and Methods

### 4.1 Data acquisition

**Fig. 3:**
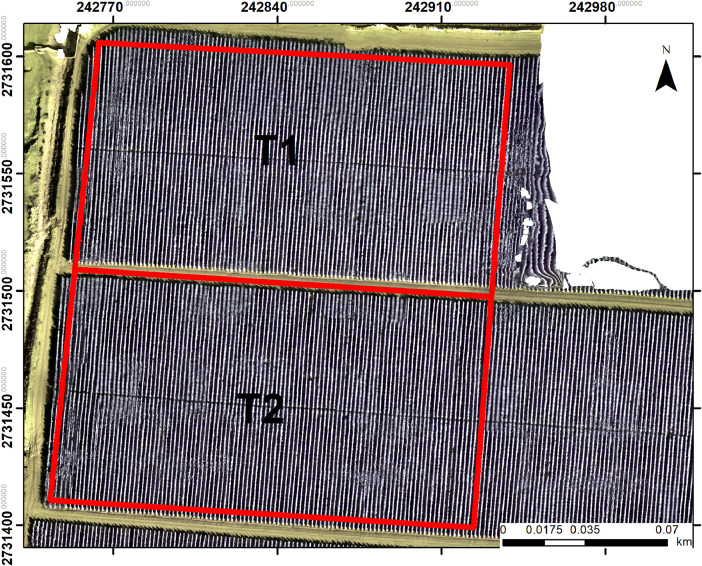
Treatments delimitation in the cherry tomato crop. T1 was cultivated using an organic-mineral fertilization strategy, as detailed in Table 4, while T2 was considered as a repetition.

The study area is in the southwestern part of the municipality of Navolato, Sinaloa, Mexico. The site is located at coordinates 24° 40′ 35.05′′ and -107° 32′ 27.99′′. The experiment was conducted from October 2021 to January 2022. The temperature ranged from 34 °C to 13 °C. The cherry tomato crop was divided into treatments (T1 and T2) as seen in Fig. 3.

The photogrammetric flight plan was designed to obtain images with a pixel size equal to or smaller than 2 cm. A flight speed of 14.1 m/s and a flight height of 40 m were used in all photogrammetric flights. The camera was configured separately considering flight plan parameters and camera characteristics (Table 2). The camera configuration parameters were image size of 4000 × 3000 px, single shot mode (non-sequential), automatic shutter, GNSS antenna metadata for each of the images, and image format (RAW). The resulting images had a longitudinal and transverse overlap of 92.8 % and 62 % respectively, with photos captured every second. The resulting Ground Sample Distance (GSD) was 1.83 cm.

**Table 2:** Multispectral camera characteristics (Mapir Survey 3W).

Camera features	
Resolution	12 Mpx (4000 × 3000 px)
Lens optics	87° HFOV (19 mm) f/2.8 Aperture, −1 % Extreme Low Distortion (Non-Fisheye) Glass Lens.
GSD	5.5 cm/px a 120 m
Sensor	Sony ExmorR IMX117 12MP (Bayer RGB)
GPS/GNSS (External)	u-blox UBX-G7020-KT
Image capture interval	1.0 s

The calibration consisted of capturing a pair of images of the Ground Target before performing the photogrammetric flight. Then, these images were processed using the MAPIR Camera Control (MCC) software. MCC is an open-source software that performs the calibration using the empirical linear method that converts the pixel values to calibrated reflectance percentages through a linear regression analysis using known reflectance values [24]. MCC consists of importing the images in RAW format. Then, the radiometric calibration is performed using the Ground Target images. As a result, TIF-processed images are generated as pixel value (DN) or reflectance percent value (%).

The processing and generation of orthomosaics was carried out in AgisoftMetashape professional version 1.5.1 based on Structure from Motion (SfM) algorithms for photogrammetry. The workflow consisted of six stages. The first stage consisted of estimating the camera position and its external orientation. Then, a sparse point cloud model was built and the geographic coordinate system was set. The next stage consisted of reconstructing the surface terrain through the generation of a dense point cloud. By interpolating the dense point cloud, a digital elevation model (DEM) was obtained. Finally, the orthomosaic was generated based on the external orientation of the image and the DEM surface. The orthomosaic is presented in '.TIF' format with a file size between 3.44 and 1.82 GB. Table 3 shows the parameters used for digital photogrammetric processing.

**Table 3:** Parameters used for digital photogrammetric processing.

Workflow	Parameters	Setting
Camera alignment	Accuracy	Low/Medium
	Generic preselection	No
	Reference preselection	Yes
	Key point limit	40,000
	Tie point limit	10,000
	Adaptive camera model fitting	Yes
Dense point cloud generation	Quality	High
	Depth filtering	Aggressive
DEM generation	Source data	Dense cloud
	Interpolation	Enabled
Orthomosaic generation	Surface	DEM
	Blending mode	Mosaic
	Hole filling	Yes

**Fig. 4:**
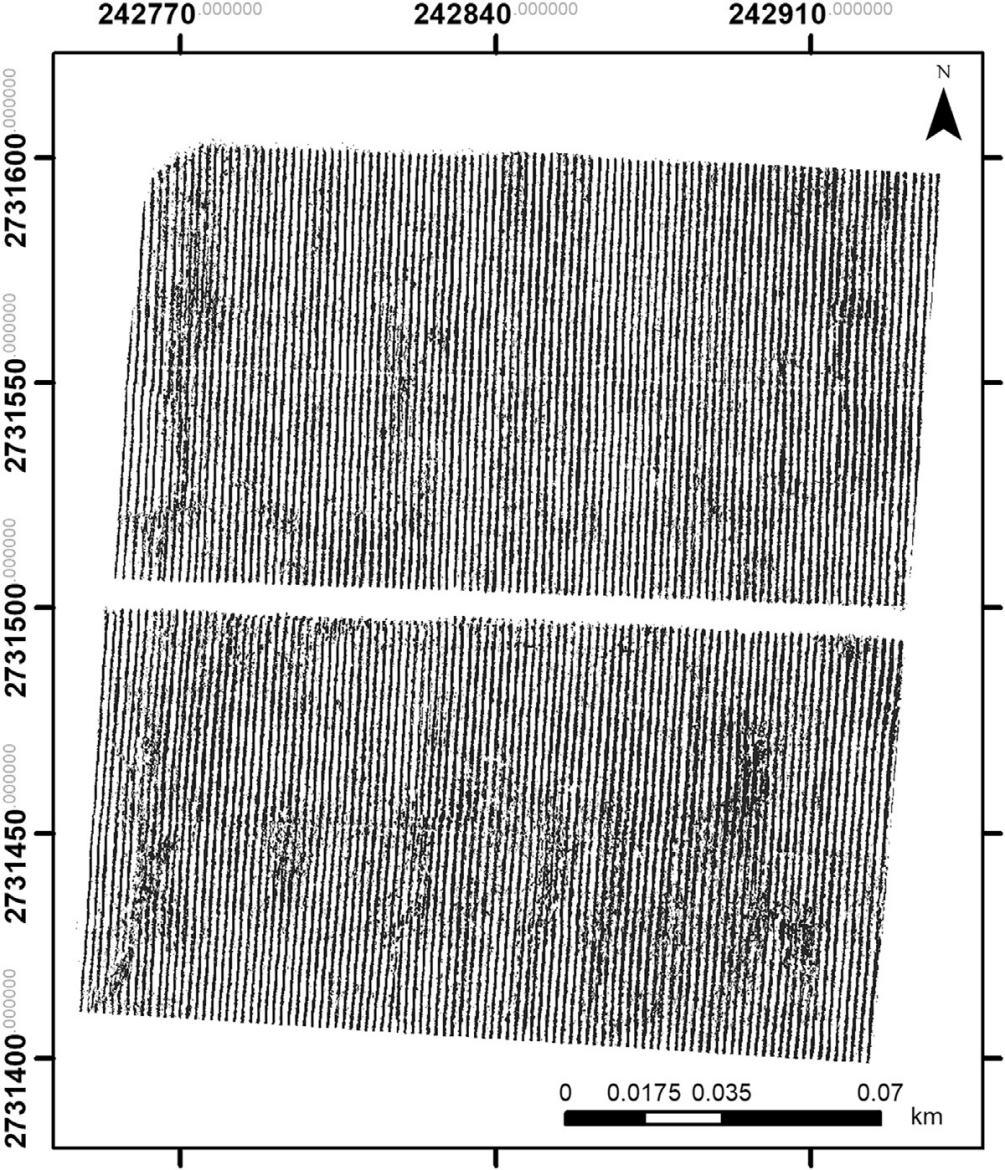
Example of the orthomosaic obtained when using the binary mask. A photogrammetric flight was carried out on November 10th 2021.

The binary mask (Fig. 4) was obtained with a supervised classification in ENVI software (version 5.3). The mask was used to reduce noise caused by shadow and ground pixels. In turn, this situation would reduce the uncertainties that could affect the geospatial analysis of the agricultural crop. Hence, two photogrammetric products (orthomosaics) were obtained (when using and without using the binary mask). Both orthomosaics are provided in the dataset.

### 4.2 Experiment design, materials, and methods

The outdoor cultivation of cherry tomatoes started on September 6, 2021, once the tomato seeds were germinated in a greenhouse. The crop was managed locally supporting and training the tomato plants with nylon twine. This management helps to keep the plants upright, preventing them from falling over or breaking under the weight of the fruit and ensuring that the plants are well-maintained and organized. The study area was divided into two open fiel parcels (T1 and T2). T1 was based on an organic-mineral fertilization (Table 4), while T2 was considered as a repetition. The detailed information of the agricultural crop conditions are given in Chávez-Martínez et al. [1]. It should be noted that there was no induced stress in any treatment.

**Table 4:** Composition of the organic-mineral fertilizer supplied.

Active ingredients and composition	Weight (%)
Magnesium	1.5
Sulfur	4.0
Boron	0.16
Iron	3.5
Manganese	0.75
Molybdenum	0.004
Zinc	0.76
Humic acid	0.54
Inert ingredients	88.045
Total	100

A DJI Phantom 4 Pro UAV was used for the photogrammetric flights. A Mapir Survey 3W multispectral camera was mounted on this UAV to obtain images in the RGN spectral range. The DJI GS Pro application was used to define the route (polygon), speed, and flight height (Table 5). The photogrammetric flight parameters were constant for all flights performed. These parameters were set according to the resolution of the multispectral camera sensor system. All photogrammetric flights were performed under nadir conditions.

**Table 5:** Photogrammetric flight parameters and image features.

Flight parameters		Image features	
Speed	14.1 m/s	Image angle	Nadir (90°)
Altitude	40 m	GSD	1.83 cm
GSD	1.00 cm	Longitudinal Overlap	92.8%
Image angle	Nadir (90°)	Transverse Overlap	62%

The camera captured the images in RAW + JPG format, which were used for the radiometric calibration. This radiometric calibration process consisted of capturing an image of the Ground Target prior to flight. The images were processed with MCC software using the empirical line method [24]. This method consisted of converting the radiation obtained by the camera sensor into reflectance through regression analysis using known reflectance values of Ground Target. As a result of this image processing, images were obtained in TIF format with a radiometric resolution of 12 bits. The order of image channels is as follows: Channel 1, or red (reflected red light); Channel 2, or green (reflected green light); and Channel 3, or blue (reflected near-infrared light). These images are the ones provided in the dataset. Table 6 shows some details about the conditions of the photogrammetric flights: date, hour, temperature, and humidity relative. In addition, some observations are provided to identify the phenological stage of the plant (BBCH scale). The first flight was carried out when the plants were more than 15 days after being transplanted from the greenhouse to outdoor cultivation.

**Table 6:** Photogrammetric flight logs.

Flight	Date	Hour	Weather conditions	Temperature in °C	Humidity Relative	BBCH scale	Notes
V1	15/10/2021	8:00 AM	Sunny	23.5	70 %	20–29	The plant is developing foliage
V2	24/10/2021	8:00 AM	Sunny	22.3	81 %	50–59	The plant starts to produce flowers.
V3	10/11/2021	8:00 AM	Sunny	20.2	68 %	60–69	The development of fruit is observed
V4	21/11/2021	7:30 AM	Sunny	19.5	67 %	70–79	The plant is a fruit-bearing crop
V5	03/12/2021	11:15 AM	Sunny	28.5	57 %	70–79	The maturation of some tomatoes is observed. The size and color of the fruit continue to develop.
V6	18/12/2021	8:30 AM	Sunny	19.8	69 %	80–89	Most of the tomatoes are fully mature and ready for harvest.
V7	23/01/2022	9:00 AM	Moderate cloud cover	18.5	63 %	90–99	The plant begins to deteriorate. Leaves start turning yellow.

The Jeffries-Matusita (JM) distance (Table 7) between pairs of objects analysis was applied to this dataset [1]. This spectral separability analysis quantifies the probability distributions of each object based on their spectral signatures. When the spectral signatures of the different sampled objects are identical, the JM distance tends to 0, while a JM distance of 2 indicates that the two objects are perfectly separable spectrally [25,26]. For this dataset, six pairs of objects were identified: Plastic-Crop, Soil-Shadow, Shadow-Crop, Soil-Crop, Soil-plastic, and Shadow-Plastic, resulting in a spectral distance very close to 2 in all cases [1], indicating that all these objects can be classified reliably. Based on this analysis, ground truth data is not essential for digital classification since objects present in the dataset are spectrally different.

**Table 7:** Variation in JM distance values across pair categories.

Flight date	Classes	JM distance
15/10/2021	Plastic-Crop	1.73
	Soil-Shadow	1.77
	Shadow-Crop	1.93
	Soil-Crop	1.95
	Soil-Plastic	1.96
	Shadow-Plastic	2.00
24/10/2021	Plastic-Crop	1.93
	Soil-Shadow	1.76
	Shadow-Crop	1.94
	Soil-Crop	1.98
	Soil-Plastic	1.98
	Shadow-Plastic	2.00
10/11/2021	Shadow-Crop	1.96
	Soil-Shadow	1.99
	Soil-Crop	2.00
21/11/2021	Shadow-Crop	1.93
	Soil-Shadow	2.00
	Soil-Crop	2.00
03/12/2021	Shadow-Crop	1.89
	Soil-Shadow	2.00
	Soil-Crop	1.99
18/12/2021	Shadow-Crop	1.91
	Soil-Shadow	2.00
	Soil-Crop	2.00
23/01/2022	Shadow-Crop	1.90
	Soil-Shadow	2.00
	Soil-Crop	2.00

## Limitations

The multispectral dataset acquired via UAV is subject to various limitations:

- The multispectral sensor is limited to green, red, and near-infrared bands. Therefore, the analysis of crop health and productivity is restricted to a limited set of vegetation indices.
- This study is also limited by the lack of ground-truth data on plant properties (biomass, height, and chlorophyll content) and soil moisture. Including such data would allow correlation with spectral responses, leading to more robust analysis and conclusions regarding crop health and productivity.
- Another key limitation is related to data acquisition. Maintaining a consistent flight altitude is crucial for uniform Ground Sampling Distance across the entire dataset. Even in the absence of significant altitude variations within the study area, uneven terrain could negatively affect spatial resolution, resulting in inconsistencies in data quality.
- Swath width of the multispectral camera determines the overlapping flight lines to achieve sufficient coverage. In this study, a high longitudinal and transverse overlap was considered for the dataset, which increased flight time and data processing complexity.
- Limited battery life of the UAV restricted the area covered in a single flight. For this dataset, we planned three flights because of battery changes, adding complexity and potential for inconsistencies between datasets.
- Other limitations could be related to external factors such as wind, clouds, or sunlight. These external factors could lead to positional inaccuracies or image blurring, decreasing the image quality. The presence of strong winds or rain could even postpone the flight mission.
- For this dataset, multispectral images required significant storage capacity. The ability to store and subsequently transfer large datasets also needs to be considered.

## Ethics Statement

The dataset does not include animal in experiments, human subjects or data collected from social media.

## CRediT Author Statement

O.C.M., S.A.M.A., J.G.R.P.: Conceptualization; O.C.M., S.A.M.A., and J.G.R.P.: Methodology; O.C.M., S.A.M.A., A.J.S.G and Z.D.M.F.: Validation; O.C.M., S.A.M.A., and J.G.R.P.: Formal analysis; O.C.M., S.A.M.A., J.G.R.P., and Z.D.M.F.: Investigation; S.A.M.A.: Resources; O.C.M., S.A.M.A., J.G.R.P., Z.D.M.F., and A.J.S.G: Data processing; O.C.M., S.A.M.A., and J.G.R.P.: Writing; S.A.M.A., and J.G.R.P.: Review and editing of the draft; Z.D.M.F. and A.J.S.G.: Visualization; S.A.M.A., and J.G.R.P.: Supervision; All authors have read and accepted the published version of this manuscript.

## Funding

This research did not receive any specific grant from funding agencies in the public, commercial, or not-for-profit sectors.

## Data Availability

Dryadtomatodb.zip (Original data).
